# Advanced clinical services in community pharmacies: training challenges and real-world applications – a focus on Greek-speaking countries

**DOI:** 10.1080/20523211.2025.2455066

**Published:** 2025-01-29

**Authors:** Aliki Peletidi, Michael Petrides, Vasilis Birlirakis, Diamantis Klimentidis, Konstantinos Liaras, Christos Petrou

**Affiliations:** a Pharmacy Programme, Department of Health Sciences, School of Life and Health Sciences, University of Nicosia, Nicosia, Cyprus; b School of Pharmacy, Faculty of Medical Sciences, Newcastle University, Newcastle upon Tyne, UK; c Bioactive Molecules Research Center (BioMoReC), University of Nicosia, Nicosia, Cyprus; d President of the Federation of the Cooperative Pharmacists of Greece, Community Pharmacist, Volos, Greece; e Clinical Pharmacist, Psychiatric Clinic ‘Agia Aikaterini’, Thessaloniki, Greece

## 1. Background

The role of pharmacists in primary healthcare is gaining increasing recognition. As providers of primary healthcare, pharmacists play a crucial role in medication management, disease treatment, and health promotion/prevention. One of their core responsibilities is to ensure the safe and effective delivery of care. Community pharmacists already make a significant, sustainable impact on the health of the individuals and communities they serve. Both the Pharmaceutical Group of the European Union (PGEU) and the International Pharmaceutical Federation (FIP) envision community pharmacists as essential health professionals who contribute dynamically and sustainably to individual and community health while also bolstering health systems.

As healthcare professionals, pharmacists must differentiate themselves from retail stores. For this reason, the evolution of the pharmacy into a primary care centre – through its services – is a step towards better health outcomes, greater trust, and loyal patients/visitors. The evolution of the pharmacy into a primary care centre – through its services – represents a significant area of novelty in healthcare. This transition is crucial as it positions pharmacists as primary healthcare providers, bridging the clinical role of the community pharmacy with the well-being of the community. Such integration allows pharmacists to take on expanded roles in disease management and health promotion, thereby alleviating pressure on traditional healthcare settings. Pharmacists, as healthcare professionals, are an integral part of primary health care, and this should be advocated in the minds of public and policymakers, according tο the International Pharmaceutical Federation (FIP) latest awareness campaign (May 2024) named ‘Think Health, Think Pharmacy’ (International Pharmaceutical Federation-FIP, 2024).

Community pharmacists work at the heart of communities, providing high-quality professional advice on the safe, effective, and rational use of medicinal products. Often, they are the first and last point of contact between the patient and the health system. As such, they contribute invaluable to the health of more than 500 million people across Europe. Moreover, the FIP for the World Pharmacists Day 2023 sent a clear message: ‘Let pharmacies do more’. This aligns with the Organisation for Economic Co-operation and Development (OECD), which states in a recent publication that pharmacists could gain more expanded responsibilities and a more substantial role due to the dramatic pressures on health systems in developed countries in the coming years. Specifically, the increase in health spending (OECD, 2024) is projected to be double that of the state budgets of OECD member states (2.6% versus 1.3%), while the number of insured and retirees is significantly increasing, and staff shortages are already visible. ‘The steep increase in demand for medical care will create a lot of stress and overwork for workers in the sector. Money will not be sufficient to hire new scientists’, notes Chris James, an OECD economist specialising in health issues. In this context, the expert suggests: ‘governments could save money by rationalising the role of health professionals’.

According to the European Society of Clinical Pharmacy (ESCP), the field of Clinical Pharmacy has seen consistent and progressive growth across numerous European countries. ESCP defines clinical pharmacy as

a discipline committed to optimising the use of medicines through both practice and research to achieve person-centred objectives, addressing both individual patient-centred and wider public health goals. It is applicable in various settings (e.g. community pharmacy, etc.), it is provided exclusively by the pharmacy team, and encompasses pharmaceutical care while extending beyond it. (Dreischulte et al., 2022)

### 1.1. Pharmacy-led advanced clinical services

Pharmacists’ roles have evolved beyond dispensing medicinal products to encompass comprehensive care, including preventive and disease management services. This shift not only enhances patient care but also reduces pressure on other healthcare sectors by proactively managing patient health within community settings. Pharmacy-led services, initiated and delivered by pharmacists, aim to improve patient outcomes. Pharmacy-led services refer to healthcare activities initiated and conducted by pharmacists with the aim of improving patient health outcomes. These services are broadly categorised into preventive and disease management, each crucial to primary care, where pharmacists are increasingly recognised as essential healthcare providers. Preventive services (e.g. vaccinations, screening programmes, smoking cessation programmes, health education and promotion interventions) in pharmacies are designed to prevent illness or detect health issues at an early stage before they require more complex medical intervention Disease management services (e.g. medication therapy reviews, chronic disease management, medication adherence management, etc.) focus on providing care and support for patients with chronic conditions to improve their quality of life and prevent complications. Additionally, in numerous countries pharmacy services have been introduced and are frequently remunerated by national healthcare systems (Crespo-Gonzalez et al., 2021; Hussain & Babar, 2023). These services include medicines optimisation and medication management to improve patient outcomes. The issue of sub-optimal medication use in chronic diseases has been acknowledged for a long time. However, it is only in recent years that medicines optimisation has become a focus for policymakers in Western developed nations. A significant aspect of the government's strategy to address this involves expanding the role of community pharmacies and leveraging the specialised skills and knowledge of pharmacists related to medications (Babar, 2024).

### 1.2. The case of the United Kingdom and Germany

In Germany, community pharmacies are an integral part of the healthcare system and play a critical role in dispensing medicinal products and providing health advice to the population. To ensure the compensation of pharmacy services, the government has created a comprehensive list of reimbursable drugs and services known as the ‘Pharmaceutical Directive’. A promising example for the future of Clinical Pharmacy Services (CPS) in Europe is the recent success of the Federal Union of German Associations of Pharmacists (ABDA) announced in July 2022 (Pharmacies in Europe ABDA, 2023).

After long and difficult negotiations, the reimbursable services include: 1. Blood pressure monitoring in hypertension, 2. Ensuring proper technique in the use of inhaled drugs for patients starting new treatment or changing devices, 3. Medication review for: (a) patients on polypharmacy (≥5 drugs), (b) patients taking oral anticancer drugs, (c) patients taking immunosuppressants post-transplantation. This marks a significant change and begins a new era for community pharmacies (Schulz et al., 2023; Seidling et al., 2017; Seidling et al., 2021). Germany achieved the reimbursement of pharmacy services within community pharmacies through a combination of government regulations and healthcare policies. It is worth noting that over the past two decades various other countries have implemented and reimbursed pharmacist-led Medication Review (MR) programmes in primary care environments.

Globally, these services are known by different names, including Medicines Use Review (MUR) in the United Kingdom, MR in Denmark, and MR conducted by clinical pharmacist consultants in Slovenia. In the United States, it is referred to as Medication Therapy Management, while in Canada, it is called MedsCheck. In Australia, similar services are termed Clinical Medication Review (CMR) and Home Medication Review (HMR), among other designations (Grindrod et al., 2013; Jokanovic et al., 2015; Latif, 2018).

The German healthcare system is based on a social health insurance model, where everyone is required to have health insurance coverage. Furthermore, community pharmacies in Germany are encouraged to participate in medication management programmes, where they work closely with doctors and other healthcare providers to optimise pharmaceutical therapy for patients. These programmes aim to improve patient outcomes and reduce healthcare costs by ensuring the safe and effective use of medicinal products. Overall, Germany's approach to reimbursing pharmacy services within community pharmacies is based on recognising the significant role that pharmacists play in the healthcare system. By providing financial support and integrating pharmacies into the broader healthcare infrastructure, Germany has managed to ensure access to quality pharmacy services for its population (Eickhoff et al., 2021).

In the UK, pharmacists are expanding their clinical role within primary care, significantly enhancing pharmacy services. Traditionally focused on dispensing medications, pharmacists now provide clinical advice, vaccinations, consultations, and management of chronic conditions like diabetes and asthma. This transition capitalises on pharmacists’ expertise in medication management and patient care, integrating them into primary care settings such as GP practices and walk-in centres. Within these settings, pharmacists conduct medication reviews, offer consultations for minor ailments, and manage chronic diseases, contributing directly to improved patient outcomes and public health (McCarthy & Luke, 2019). Initiatives like the Pharmacy First service further leverage pharmacists’ skills, allowing them to treat minor ailments and reduce GP workload, thus positioning pharmacists as key healthcare providers within the community. The United Kingdom's National Health Service (NHS) is based on the principles of universality, comprehensiveness, and free access to healthcare at the point of delivery (Chopra et al., 2022). Established in 1948, the NHS aims to provide high-quality medical care to all UK residents, funded mainly through taxation. It operates under the core principles of being available to everyone, without regard to their ability to pay, and ensuring that care is based on clinical need rather than financial capacity. The NHS contracts with community pharmacies and pharmacists through a structured framework that enables the delivery of pharmacy services to the public. This contractual relationship is critical in integrating pharmacists into the broader healthcare system and expanding their role in providing essential services. The contracting process is often negotiated through representative bodies such as the Pharmaceutical Services Negotiating Committee (PSNC), ensuring that the agreements meet national health priorities while supporting the business viability of pharmacies. This contractual framework supports pharmacists in adopting clinical roles and enhances their ability to contribute significantly to patient care within the NHS (Community Pharmacy England, 2024; Reflecting on 75 years of the NHS, n.d.).

## 2. National Health Systems in Greece and Cyprus

Greece's National Health System, known as ‘Ethniko Systima Ygeias’ (ESY), was established in 1983. It is a universal system intended to provide comprehensive healthcare services to all residents. The system is funded through taxation, social security contributions, and co-payments for certain services and medicinal products. ESY provides a range of healthcare services through public hospitals, health centres, and clinics. Primary healthcare is also delivered through PEDY (Primary National Health Network) units. Despite facing significant challenges, including funding and resource constraints exacerbated by economic crisis, the system strives to maintain access to healthcare for all residents (Lionis et al., 2015; Myloneros & Sakellariou, 2021).

In June 2019, Cyprus launched the General Health System (GHS or GeSY), a comprehensive national healthcare system designed to provide all residents with equal access to healthcare services. This system is characterised by universal population coverage, ensuring that everyone is included. GeSY guarantees equal and fair treatment for all beneficiaries, allowing access to a wide range of healthcare services. Beneficiaries have the freedom to choose their healthcare providers, reflecting an emphasis on social reciprocity. It is funded through contributions from taxpayers, employers, and the Government, ensuring that healthcare services are available to everyone at the point of delivery without significant direct charges. Under the GHS, residents can choose their healthcare providers, including general practitioners and specialists, within the network. The system covers a wide range of medical services, including primary, secondary, and tertiary care, as well as prescribed medications (Petrou, 2021).

### 2.1. Community pharmacies in Greece and Cyprus

In Greece, community pharmacies are primarily funded through the social insurance system, where pharmacies are reimbursed by the National Organisation for Healthcare Provision (EOPYY) for dispensing medicinal products to insured individuals. Patients typically pay a percentage of the medication cost as a co-payment, except for certain groups (like low-income or chronic patients) who are eligible for reduced or no co-payments (Foundation for Economic & Industrial Research, 2018).

In Cyprus, under the General Health System (GeSY) plan, pharmacists may provide various healthcare services to beneficiaries, including dispensing prescriptions, offering information and advice on the safe and responsible use of medicinal products, and providing generic substitution for the least expensive pharmaceutical product of the same active substance and form. Currently dispensing is the only service reimbursed (Petrou, 2021). GeSY covers only prescription only medicines (POM). These medicinal products are listed in the Pharmaceutical Product Catalogue, prepared by the Health Insurance Organization (HIO) upon recommendation from the relevant scientific committee. Over the counter medications, like analgestics (e.g. paracetamol), and cold remedies (e.g. cough syrups), as well as vitamins are not covered by GeSY. However, some exceptions apply such as Vitamin D for patients with osteoporosis, those on long-term corticosteroid therapy, and patients with thalassaemia and aspirin which is also provided to patients for secondary prevention of cardiovascular diseases, specifically for those who have already experienced a myocardial infarction or similar event, to help prevent a recurrence. Such products are available through community pharmacies and reimbursed by GESY. If a beneficiary chooses a more expensive medicinal product than what GeSY fully covers, they pay a ‘Contribution II fee’. However, there is at least one medicinal product per category for which beneficiaries are exempt from Contribution II. Specific regulations apply to prescribing certain medicinal products, such as protocols/guidelines, prescriptions from specific medical specialties, or pre-approval by the system (Petrou, 2021).

Today, the face of the community pharmacy in both countries does not promote the modern role of the community pharmacist as recorded in the FIP's Development Goals. The pharmacy prominently displays its commercial character, treating the visitor as a customer and consumer of preparations and para-pharmaceutical products. Conversely, the FIP report states that sales are just a small part of the services that the pharmacy of 2030 is called to provide, while the person-centred approach to the visitor's needs and the provision of primary healthcare services are the main demands. The goals of the FIP for the community pharmacy of 2030 outline services such as health promotion, prevention, and disease management services that require very different spatial interpretations from those prevailing in the reality of Greece and Cyprus. Based on the new revised roles of the pharmacist, the philosophy of pharmacy operation must change.

Interestingly, Greece has the highest pharmacy density within the European Union (EU), with 97 pharmacies per 100,000 inhabitants, compared to the EU average of 32 pharmacies per 100,000 inhabitants (2023). Cyprus ranks second, with 63 pharmacies per 100,000 inhabitants. In Cyprus, the average number of pharmacists per pharmacy is 2.67. However, in Greece the average number of pharmacists per pharmacy is 1.05, indicating a shortage of pharmacy staff. It is important to note that to provide clinical pharmacy-led services effectively, pharmacists need not only proper training but also sufficient time to deliver these services, necessitating more pharmacists per community pharmacy (International Pharmaceutical Federation-FIP, 2021).

Patient-centred services in the community pharmacies in Greece are a relatively recent development. Historically, community pharmacies in Greece were primarily focused on traditional activities, such as filling prescriptions and dispensing non-prescription medicinal products. The first significant change occurred in 2017, when community pharmacies in Greece were officially recognised as healthcare providers within the context of Primary Care Networks (Greek Government, 2017). Two years later, following the decision of the Minister of Health and based on the opinion of the Panhellenic Pharmaceutical Association, licensed and certified community pharmacists (after completing relevant training and accreditation programmes in Greece (as specified in paragraph 1 of article 160 of law 4600/2019 (A’ 43) and the decision ‘Γ5α/Γ.Π.οικ.49734/2.7.2019’ by the Minister of Health), are authorised to administer all vaccinations listed in the National Adult Vaccination Programme, as well as the SARS-CoV-2 coronavirus vaccine prescribed by doctors, operating as Primary Health Care Units (Ν. 5102/2024 (ΦΕΚ A 55 – 13.04.2024). However, this excludes the administration of live attenuated virus vaccines to immunocompromised patients and all vaccines except the flu vaccine to pregnant women. Later that year, a ministerial decree designated the Department of Social and Family Medicine of the Medical School at the University of Crete to develop the training programme and serve as the accreditation body for community pharmacists (Greek Government, 2019a, 2019b). Both the training and the examination of candidates were conducted online, facilitated by the Panhellenic Pharmaceutical Association.

The COVID-19 pandemic significantly expanded pharmacy-based services. Initially, pharmacies were designated as centres where citizens could schedule COVID-19 vaccination appointments (Greek Government, 2020). Shortly thereafter, pharmacies were authorised to conduct formal COVID-19 rapid antigen testing and update the national registry with the testing results (Greek Government, 2021). These services proved successful in terms of patient satisfaction, prompting the Greek government to enhance the role of community pharmacies further. In the most recent development, licensed pharmacists have been authorised to administer COVID-19 vaccines (Greek Government, 2024).

In 2024, the National Prevention Programme ‘*ΠΡΟΛΑΜΒΑΝΩ’* for colorectal cancer is in full swing in Greece, as part of the broader ‘National Public Health Prevention Programme “Spyros Doxiadis”’, funded by the European Union through NextGenerationEU. Its aim is the early diagnosis and treatment of colorectal cancer and the promotion of screening through an organised, structured and free programme for citizens (National Programme for the Prevention of Colorectal Cancer, 2024). The programme targets all citizens aged 50–69 who have a Social Security Number (AMKA), regardless of their insurance status. Eligible individuals receive informative SMS messages and can obtain free self-tests from participating pharmacies. In the event of a positive result, a free visit to a gastroenterologist is provided, and if deemed necessary, a diagnostic colonoscopy is performed (National Programme for the Prevention of Colorectal Cancer, 2024).

In contrast, community pharmacists in Cyprus primarily focus on dispensing prescribed and over the counter medications. However, during COVID-19 pandemic, pharmacies in Cyprus were authorised to conduct formal COVID-19 rapid antigen testing and update the national registry with the testing results.

Pharmacists in Greece, apart from the above mentioned, are offering some opportunistic interventions in their community pharmacies (e.g. blood pressure measurement, etc.). However, structured advanced clinical services in community pharmacies provide a more reliable, efficient, and professional approach to patient care compared to opportunistic services, leading to better health outcomes, enhanced pharmacy operations, and a stronger healthcare system. This is crucial for maintaining high-quality service across different patients and conditions, reducing variability in treatment outcomes. Additionally, by offering structured services, pharmacists can follow evidence-based guidelines and protocols that have been proven to improve patient outcomes. This systematic approach allows for better management of chronic diseases, more effective interventions, and overall enhanced patient health. Besides, structured services provide a framework for ongoing patient education and engagement, which is crucial for chronic disease management and preventive care. Individuals are more likely to understand their treatment plans and adhere to prescribed therapies when services are delivered in a consistent and structured manner. Lastly, structured programmes allow for better monitoring and evaluation of service effectiveness. This accountability ensures that services meet certain quality standards and allows for continuous improvement based on feedback and outcomes.

The expansion of structured clinical pharmacy services in Greek-speaking countries (Greece & Cyprus), after compulsory training and certification, will play a critical role in supporting the national health system in various ways. These services contribute to improving patient outcomes, increased access to healthcare, and improved efficiency. According to Peletidi et al. (2021) Greek pharmacists were found to be willing to expand their role and offer public health services (ref). In 2017, the first pilot weight management (WM) service to reduce the cardiovascular disease was designed, delivered, and evaluated in Greece. The primary goal of the WM service was a minimum weight reduction of 5% of the initial weight, while the secondary outcomes were reductions in body mass index (BMI), waist circumference (WC), blood pressure (BP), AUDIT-C score, an increase in the Mediterranean diet (MD) score and improvement in physical activity levels. This study was a 10-week weight management (WM) programme conducted in community pharmacies in Patras (third largest city of Greece). Nearly every participant who enrolled in the 20 participating pharmacies, 97.4% (*n* = 114/117), achieved the service's goal by losing at least 5% of their initial weight. The average percentage of total weight loss of the 117 participants at week 10 was 8.97% (SD 2.65) (*p* value < 0.001; 95% CI 8.48-9.45). A significant reduction in the waist-to-height ratio (WHtR) was observed in both men (*p* value = 0.004) and women (*p* value < 0.001). The BP and AUDIT-C scores and the levels of physical activity of the participants significantly improved (*p* value < 0.001), as did their MD scores. This study provided the first evidence that Greek pharmacists have the potential to play a significant role in primary care and that after training, they can provide public health services both for the benefit of the public and to enhance their clinical role.

### 2.2. Designing training to prepare future and current pharmacists to offer advanced clinical services

According to Scahill et al. (2017), pharmaceutical education is divided into four sectors: Pharmaceutics (sometimes referred to as Pharmaceutical Technology) and Pharmacokinetics, (2) Pharmaceutical Chemistry (including chemistry of natural products), (3) Pharmacology, and (4) Pharmacy Practice which includes clinical pharmacy, pharmacotherapy, social and administrative sciences, and pharmaceutical care.

As of the latest updates, most European countries have integrated both scientific and clinical modules in their pharmacy degree programmes (MPharm or BPharm). This integration reflects the evolving role of pharmacists in healthcare systems, where a combination of deep scientific knowledge and clinical skills is essential for effective practice. The classification of pharmacy degrees as ‘science-based’ or ‘clinical-based’ can vary depending on the specific educational structure and curriculum focus of each country.

The Pharmacy Programme (MPharm) at the University of Nicosia (University of Nicosia, 2024) aligns with the modern challenges of pharmaceutical education since 2013 and offers, in addition to the basic pillars of pharmaceutical education, clinical courses in its curriculum. The recently accredited curriculum includes clinical courses from the second year of study (Introduction to Pharmacy Practice, Public Health, Primary Health Care), and continues with advanced clinical courses in the 4th year of study: Pharmacy Practice I & II and Social Pharmacy). Additionally, the University of Nicosia's Pharmacy Programme has created the first Pharmacy Practice Lab/Simulation Pharmacy, unique in Greece and Cyprus which was designed based on the modern specifications of the FIP for the 2030 pharmacy, as a primary healthcare centre (Pharmacy, 2030–PGEU, 2019). The Pharmacy Practice lab is a fundamental component of pharmaceutical education, as it operates as a practical learning environment where future pharmacists (students) can apply theoretical knowledge and develop practical skills necessary for their future career as pharmacists. The value of the pharmaceutical practice lab lies in its ability to bridge the gap between conventional classroom teaching and training and practical exercise in the real world using experiential learning. Applied Pharmacy Practice (including all the above) is a significant branch in the practice of pharmacy – it has much to offer and has played a significant role in transforming the profession of the pharmacist over the last two decades. The fourth sector of pharmacy (i.e., Pharmacy Practice) is part of the ‘re-professionalisation’ agenda in both the hospital and community sectors of pharmacy. Also, Pharmacy Practice is generally a sector of pharmacy that provides students with excellent consulting skills and clinical knowledge.

The integration of pharmacy practice courses into the first cycle of MPharm studies at universities in Greece increasingly recognised as an essential step to prepare future pharmacists for the modern challenges and demands of the profession in the twenty-first century. As the role of the pharmacist transforms, advanced clinical skills and the promotion of public health are becoming central components of their responsibilities. This transition positions pharmacy services that focus on patient care and health management as pivotal for the future of pharmacies by 2030 (Pharmacy, 2030–PGEU, 2019). Apart from the University of Nicosia mentioned above, many European universities have already embraced the integration of these elements into their curricula. In the United Kingdom, clinical pharmacy practice is a core component of pharmacy education. Similarly, universities in Finland, Denmark, and Belgium, such as the University of Helsinki, the University of Copenhagen, and KU Leuven, have developed programmes with an emphasis on social pharmacy and clinical care (Department of Pharmaceutical and Pharmacological Sciences, 2024; Faculty of Pharmacy University of Helsinki, 2024; University of Copenhagen, 2024). Furthermore, in Spain, the University of Barcelona offers courses like ‘Clinical Pharmacy and Pharmaceutical Care’, while Heidelberg University in Germany has introduced courses in ‘Clinical Pharmacy’. In Italy, the University of Bologna offers ‘Pharmaceutical Care’ courses, and the University of Pavia includes courses on the ‘Diagnosis and Treatment of Minor Illnesses’ (Pharmacy Università Di Pavia, 2024; Pharmacy – Heidelberg University, 2024; Pharmacy – G1051, 2024).

In contrast, Greece's Pharmacy degree (MPharm) curriculum, while incorporating some clinical aspects, still prioritises science-based modules over clinical ones. This imbalance is largely due to historical educational standards in Greece, which have traditionally emphasised pharmaceutical sciences. Moreover, the field of Pharmacy Practice has not yet been institutionalised. Consequently, there is a discernible gap in the clinical knowledge of both current practising pharmacists and future pharmacists in Greece. To address this gap and enable Greek pharmacists to effectively offer clinical pharmacy services in community settings, there is a pressing need for intensive training and curriculum reform in Greek pharmacy education.

Pharmacists in the Greek-speaking countries as well as globally at the end of their studies should be able to confidently provide safe and effective care to their patients who meets their health needs. Pharmacy Practice education is a great addition to the existing courses that current registered pharmacists have successfully completed because it will give them the opportunity (a) to gain knowledge about symptom management and minor ailments, (b) to apply into practice all the knowledge gained from the other three pharmacy sectors, which are already taught, in order to offer clinical services within the community pharmacy, (c) to learn how pharmacists can promote public health through their daily practice, and (d) to further develop their communication skills in a safe environment with the ultimate goal of better patient care.

#### 2.2.1. The need for continuous professional development programme and a revalidation scheme

In both Greece and Cyprus, the process for graduate pharmacists to achieve their registration involves several key steps that ensure they are qualified to practice pharmacy in a professional and safe manner. In both countries, after completing their practicum period (currently 12 months), graduate pharmacists need to pass a registration examination (varies between Greece and Cyprus). The examination is essential for assessing their proficiency and readiness to practice as pharmacists.

Nevertheless, continuous professional development (CPD) is crucial for community pharmacists, particularly as their clinical roles continue to expand within the healthcare system. More specifically, CPD is essential for community pharmacists to maintain their competence, adapt to new roles, and provide high-quality care. The science of Pharmacy is constantly evolving, with new treatments, medicinal products, and health care protocols emerging regularly. CPD ensures that pharmacists stay current with these developments, enabling them to provide accurate, safe, and effective care. Furthermore, CPD programmes often cover a wide range of topics, including disease management, patient counselling, and the use of new medical technologies. This broadens the pharmacists’ knowledge and skills, making them more competent in their clinical roles. Besides, regular participation in CPD activities (both planned and unplanned) could enhance pharmacists’ confidence in their clinical abilities. It also boosts their credibility both among colleagues and patients/individuals, which is important for the professional relationships that underpin safe and effective care provided.

Importantly, pharmacists have a professional duty to maintain their competence. CPD is not just beneficial for personal and professional growth. CPD should be a legal requirement to ensure that pharmacists meet the regulatory standards necessary to practice. Therefore, it is proposed that a need for a revalidation scheme every 10 years in Greek-speaking countries is essential.

The structured revalidation scheme certifies that pharmacists remain fit to practice throughout their careers, thereby safeguarding patient safety and public health. More specifically, it can ensure that pharmacists continue to meet the necessary professional standards over time. This is crucial in a profession where outdated knowledge can lead to serious errors in patient care but can also reinforce the public’s trust in the pharmacy profession by demonstrating a commitment to maintaining high standards of practice. In addition, as the role of pharmacists expands to include more direct patient care activities, revalidation ensures that pharmacists are competent in these new responsibilities, which may not have been part of their initial training as described above.

### 2.3. Proposed training programme

The proposed formal postgraduate training programme for pharmacists in Greece and Cyprus, allows community pharmacists to expand their clinical role in offering pharmacy-led public health services. This accelerated learning programme provides the essential knowledge required for community pharmacists to be able to conduct preventive and health promotion services, as well as services related to chronic diseases, within their pharmacies. This programme offers both theoretical and practical skills regarding the clinical role of the pharmacist in the community and the provision of advanced health services through it (as per Tables 1 and 2). Pharmacists have the option to enrol in either proposed Module 1 or Module 2. However, to obtain certification to provide clinical pharmacy services, they must successfully complete both modules.

**Table 1.** Proposed Module 1.

**Course Title:** Health Promotion and Preventive Services from the Community Pharmacy **Course Code:** PHSH601 **Course Type:** Mandatory **Level:** Pharmacy Graduates/Licensed Pharmacists **Total Hours:** 20 **Total Weeks:** 9 **Course Purpose and Objectives:** Preventive healthcare aims to prevent the onset of disease (primary prevention), reduce the progression of disease by detecting it before it becomes symptomatic (secondary prevention), and reduce the impact of the disease if it occurs (tertiary prevention). Community pharmacists are health professionals in primary care, who can provide prevention and health promotion services to the public. This module aims to consistently provide a wide range of health promotion interventions through community pharmacies to meet the population's needs, improving the health and well-being of the local population and contributing to reducing health inequalities. Additionally, pharmacists will acquire the knowledge and skills to provide prevention services through their community pharmacy. **Learning Outcomes:** After completing the course, pharmacists will be able to: Explain the need, objectives, and content of each provided service. Select, design, and implement the provision of each service through their pharmacy. Organise the resources of the community pharmacy for the provision of each service. Evaluate the opportunities for promoting each service using appropriate tools in the local community. Provide each service to the target population. Evaluate each of the provided services for improvement. Enhance interprofessional communication and collaborative practice to enhance the services. **Course Content:** **Week 1** 1. Basic Principles of Health Promotion, Disease Prevention and Emergency Preparedness 2. Introduction to Services Guided by the Community Pharmacy **Week 2** 3. Communication Skills for Pharmacists, Person-Centred Care, and Active Listening/Empathy 4. Self-Care Support **Week 3** 5. Inclusive Services for LGBTQ+ **Week 4** 6. Weight Management Service 7. Promotion of Healthy Lifestyles, Healthy Eating, and Physical Activity **Week 5** 8. Immunisation and Vaccination Service 9. Smoking Cessation Service **Week 6** 10. Risk Assessment for Chronic Diseases & Preventive Screening for Early Detection of Chronic Diseases **Week 7** 11. Management of Acute & Infectious Diseases 12. Microbial Resistance 13. PoC Testing in the Community Pharmacy **Week 8** 14. Practical Exercise **Teaching Methodology:** e-Learning, asynchronous & synchronous online teaching (Webex), role-playing, real-practice scenarios, group work/problem-solving, face-to-face practical teaching **Week 9** Final Exam/OSCE

**Table 2.** Proposed Module 2.

**Course Title:** Chronic Diseases and Service Provision from Pharmacies **Course Code:** PHSH602 **Course Type:** Mandatory **Level:** Pharmacy Graduates/Licensed Pharmacists **Prerequisite:** PHSH601 **Total Hours:** 20 **Total Weeks:** 9 **Course Purpose and Objectives:** Pharmacists are increasingly recognised as key providers in primary health care, offering services related to drug use and the management of chronic diseases. This module aims to enable students to acquire the necessary knowledge about chronic diseases as well as the necessary knowledge and skills to provide services related to the pharmacotherapy of chronic diseases, which they will be able to offer as healthcare professionals in the community pharmacy setting. The main objectives of the module are: Update knowledge on new and existing pharmacotherapeutic treatments for chronic diseases Solve clinical scenarios for the most common chronic diseases Develop communication skills for pharmacists in a safe environment aimed at effective patient care Optimise pharmacotherapy **Learning Outcomes:** Upon completion of this course, pharmacists are expected to: Provide services for the management of chronic diseases in the pharmacy. Critically evaluate the general scheme of service provision related to the pharmacotherapy of chronic diseases. Evaluate and apply treatment guidelines and protocols. Explain all the steps required to take a medical history. Evaluate drug interactions to minimise the occurrence of adverse effects in clinical practice in the pharmacy. Evaluate the clinical effects of safety and effectiveness of drugs. Develop knowledge, critical thinking, and skills through relevant clinical scenarios. Apply their knowledge to optimise the health and quality of life of patients with effective, safe, and cost-effective drug administration. **Course Content:** **Week 1** 1. Minor Health Issues Service: Service purpose, general protocol, and procedures (History Taking, Health Problem Assessment, Decision on Referral or Treatment Choice, Drug Information and Education, Prevention of Pharmacotherapeutic Problems) 2. Principles of collaborative practice **Week 2** 3. General aspects & pharmacotherapy for: Diabetes/Hypertension/Heart Failure/Arrhythmias/ Angina/Stroke/Coronary Artery Disease **Week 3** 4. Asthma, COPD (including correct use of inhaled medicinal products) **Week 4** 5. Osteoporosis/Osteoarthritis/Gout/Rheumatoid Arthritis **Week 5** 6. Chronic Kidney Failure/Chronic Liver Failure/Thyroid Disorders **Week 6** 7. Patients Perspectives 8. Patient Education for Chronic Diseases 9. Management and Optimisation of Pharmacotherapy 10. New Drug Service/ Medication Adherence Service **Week 7** 11. Administration of prescribed medications for Chronic Diseases: Service purpose, general protocol, and procedures (Drug Information and Education, Prevention of Pharmacotherapeutic Problems, Monitoring of Pharmacotherapy) **Week 8** 12. Practical Exercise **Teaching Methodology:** e-Learning, asynchronous & synchronous online teaching (Webex), role-playing, real-practice scenarios, group work/problem-solving **Week 9** Final Exam/OSCE

The training is designed based on the specific educational needs of pharmacists, incorporating both lectures and active learning methods. The duration of the lectures was intentionally limited for two primary reasons: firstly, due to the significant time constraints faced by pharmacists, and secondly, to provide participants with the chance to engage in discussions about pharmacy-led clinical services with both the instructor and their colleagues. Experiential learning techniques, such as role-playing, are employed as they facilitate interaction among professionals. Furthermore, role-playing was utilised to encourage pharmacists to develop problem-solving skills using a patient-centred approach, enhance their communication abilities through dialogue, and boost their self-confidence by assimilating new knowledge acquired during this training. Additionally, empathy and emotional intelligence are advanced with peer role-play simulation interventions (Deniz et al., 2024; Hopkins, 1993).

Moreover, it is essential to utilise expert personnel for training pharmacists in clinical services for enhancing their capabilities, ensuring high standards of patient care, and adapting effectively to the expanding scope of pharmacy practice in community settings. Expert academics will bring a high level of knowledge and experience that is essential for pharmacists to develop the necessary skills to deliver clinical services effectively. These experts are typically well-versed in the latest clinical practices and medical guidelines, ensuring that the training is both current and relevant.

Upon completion of training, it is important to assess both the immediate and long-term effects (6 months after the completion of the training) of the proposed postgraduate training for pharmacists. This assessment should be conducted using an evaluation questionnaire designed to measure knowledge retention immediately after the training as well as six months later, recognising that knowledge can diminish over time. These evaluations are supported by Gass and Curry's findings (1983), which examined the retention of knowledge and skills in other health care professionals including physicians and nurses following a training. Their study found a significant decline in retained knowledge and skills six months post-training, with further reductions in basic life support abilities occurring after one year. For healthcare professionals including pharmacists, this underscores the necessity of ongoing education and refresher courses to maintain proficiency and ensure the continued delivery of high-quality care. This approach could help healthcare professionals stay updated with current practices and minimise the natural decline in skills and knowledge over time (Peletidi & Kayyali, 2022).

#### 2.3.1 Proposed modules

The proposed training programme aims to improve pharmacists’ competencies including their leadership and communication skills, their professionalism, and their clinical and critical thinking. Moreover, the proposed modules are in line with the following FIP Development Goals 2020: **1** Academic capacity, **4** Advanced and specialist development, **5** Competency development, **6** Leadership development, **7** Advancing integrated services, **8** Working with others, **9** Continuing professional development strategies, **14** Medicines expertise, **15** People-centred care, **18** Access to medicines, devices & services, **19** Patient safety and **21** Sustainability in pharmacy (FIP Development Goals – Transforming Global Pharmacy, 2020).

## 3. Conclusion

Community pharmacies are often more accessible than other healthcare facilities. By offering extended clinical services, pharmacies become a convenient access point for various health-related needs, reducing the burden on primary care facilities and emergency departments. They can also provide valuable support for preventive care through service delivery such as vaccinations, preventive health screenings, and health education. This preventive approach helps in the early detection of health risks, contributing to public health initiatives and reducing the overall burden of preventable diseases. In addition, the involvement of pharmacists in patient care can increase access to primary care and can lead to cost savings for the national health system. By preventing medication errors, improving adherence, and actively managing chronic diseases, pharmacists contribute to reducing the financial burden associated with healthcare complications that can be prevented. Currently in Greece, no formal organisation offers post-academic education to pharmacists. To improve the clinical practice of pharmacists, there is a need for postgraduate training that will educate them on how to offer structured clinical pharmacy services (both preventive and disease management services). Despite the high density of pharmacies in these countries, the opportunity to expand structured clinical pharmacy services remains largely untapped. This calls for robust evaluation strategies to assess the impact and effectiveness of proposed services. Undertaking pilot programmes, like Greece’s weight management service, can offer empirical evidence and measurable outcomes that drive the adoption of best practices.

To effectively implement clinical pharmacy services in real-world settings, it is crucial to assess the sustainability of their implementation. This assessment involves identifying both the facilitators and obstacles, including workforce challenges, government regulations, and healthcare policies. Government regulations and health policies that enhance the clinical role of pharmacists in delivering advanced services are essential for creating a more accessible, efficient, and integrated healthcare system that meets the diverse needs of the population. The Pharmaceutical Associations in both countries should collaborate with relevant Academics from the Pharmacy Practice discipline and the Ministries of Health to highlight and document the added value provided by these pharmacist-led clinical services. As the field continues to develop, ensuring that these roles are evidence-based and supported through comprehensive training and robust policy frameworks will be vital.
